# A Head-to-Head Comparison Between Plasma pTau181 and Tau PET Along the Alzheimer’s Disease Continuum

**DOI:** 10.2967/jnumed.122.264279

**Published:** 2023-03

**Authors:** Emma M. Coomans, Inge M.W. Verberk, Rik Ossenkoppele, Sander C.J. Verfaillie, Denise Visser, Mariam Gouda, Hayel Tuncel, Emma E. Wolters, Tessa Timmers, Albert D. Windhorst, Sandeep S.V. Golla, Philip Scheltens, Wiesje M. van, der Flier, Bart N.M. van Berckel, Charlotte E. Teunissen

**Affiliations:** 1 Department of Radiology and Nuclear Medicine, Amsterdam Neuroscience, Vrije Universiteit Amsterdam, Amsterdam UMC, Amsterdam, The Netherlands;; 2 Department of Clinical Chemistry, Neurochemistry Laboratory, Amsterdam Neuroscience, Vrije Universiteit Amsterdam, Amsterdam UMC, Amsterdam, The Netherlands;; 3 Alzheimer Center Amsterdam, Department of Neurology, Amsterdam Neuroscience, Vrije Universiteit Amsterdam, Amsterdam UMC, Amsterdam, The Netherlands;; 4 Clinical Memory Research Unit, Lund University, Lund, Sweden;; 5 Department of Medical Psychology, Amsterdam Public Health Research Institute, University of Amsterdam, Amsterdam UMC, Amsterdam, The Netherlands; and; 6 Department of Epidemiology and Data Science, Vrije Universiteit Amsterdam, Amsterdam UMC, Amsterdam, The Netherlands

**Keywords:** plasma pTau181, tau PET, Alzheimer’s disease

## Abstract

Both plasma tau phosphorylated at threonine-181 (pTau181) and tau PET show potential for detecting Alzheimer’s disease (AD) pathology and predicting clinical progression. In this study, we performed a head-to-head comparison between plasma pTau181 and tau PET along the AD continuum. **Methods:** We included participants from the Amsterdam Dementia Cohort who underwent ^18^F-flortaucipir (tau) PET and had a plasma sample biobanked within 12 mo from tau PET. Fifty subjective cognitive decline (SCD) participants (31 Aβ-negative and 19 Aβ-positive) and 60 Aβ-positive participants with mild cognitive impairment (MCI) or dementia due to AD were included. A subset had 2-y longitudinal plasma pTau181 and tau PET available (*n* = 40). Longitudinal neuropsychological test data covering 3.2 ± 2.7 y from both before and after tau PET were available. Plasma pTau181 and tau PET were compared in their accuracies in discriminating between cognitive stage (MCI/AD vs. SCD) and preclinical Aβ status (SCD Aβ-positive vs. SCD Aβ-negative), their associations with cross-sectional and longitudinal neuropsychological test performance, and their longitudinal changes over time. **Results:** When discriminating between preclinical Aβ status, the area under the curve (AUC) for plasma pTau181 (0.83) and tau PET (entorhinal, 0.87; temporal, 0.85; neocortical, 0.67) were equally high (all DeLong *P* > 0.05), but tau PET outperformed plasma pTau181 in discriminating MCI/AD from SCD (AUC for plasma pTau181: 0.74; AUCs for tau PET: entorhinal, 0.89; temporal, 0.92; neocortical, 0.89) (all *P* < 0.01). Overall, tau PET showed stronger associations with cognitive decline and was associated with a wider variety of cognitive tests than plasma pTau181 (plasma pTau181, −0.02 > β < −0.12; tau PET, −0.01 > β < −0.22). Both plasma pTau181 and tau PET increased more steeply over time in MCI/AD than in SCD (*P* < 0.05), but only tau PET annual changes were associated with cognitive decline. **Conclusion:** Our results suggest that plasma pTau181 and tau PET perform equally well in identifying Aβ pathology but that tau PET better monitors disease stage and clinical progression.

## Visual Abstract

**Figure:**
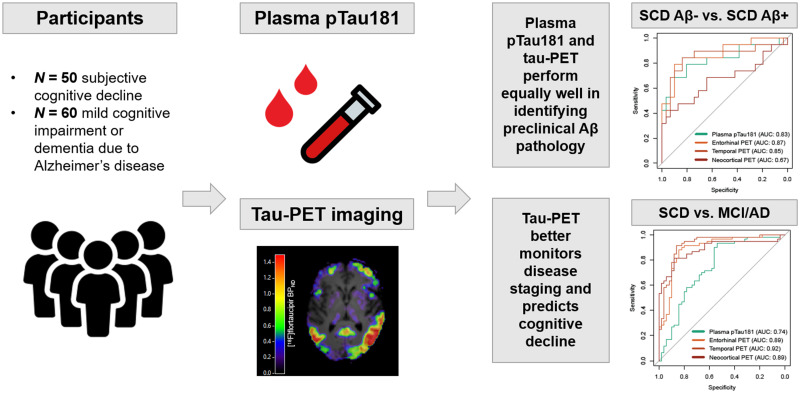

Neurofibrillary tau tangles consist of hyperphosphorylated tau (pTau) and are a pathologic hallmark of Alzheimer’s disease (AD) (1). Tau pathology in AD is closely associated with clinical symptoms and disease severity (2*,*^3^). As such, in vivo assessment of tau is expected to provide both accurate diagnostic and accurate prognostic information. Biomarkers for detecting in vivo tau pathology include pTau measurements in cerebrospinal fluid (4), imaging of tracer binding to tau paired helical filaments using PET (5), and, since a few years ago, pTau measurements in blood (6–8). Blood-based biomarkers have major advantages, including easy accessibility, wide applicability, relative noninvasiveness, and low costs and can therefore easily be repeated over time, whereas PET biomarkers, although expensive, have the advantage of providing spatial information on tracer binding throughout the brain.

Studies have shown that plasma tau phosphorylated at threonine-181 (pTau181) can discriminate AD dementia from both non-AD dementias and Aβ-negative cognitively unimpaired older adults (7–10), and can predict cognitive decline (11*,*^12^) and progression to mild cognitive impairment (MCI) or dementia (13*,*^14^). Tau PET can also discriminate between AD dementia and both non-AD dementias and cognitively unimpaired older adults (15*,*^16^), and strong associations with subsequent cognitive decline and brain atrophy have consistently been reported (17*,*^18^). Both plasma pTau181 and tau PET are closely associated with amyloid-β (Aβ) pathology (7*,*^19^). Although both tau biomarkers show potential for AD diagnosis and prognosis, head-to-head comparison studies are limited. With the recent Food and Drug Administration approval of the tau PET tracer ^18^F-flortaucipir for clinical use, and intentions for plasma pTau to eventually be used in the clinic, there is a need to compare these biomarkers to guide clinicians in performing their clinical work-up and researchers in designing trials.

The overarching aim of this study was to perform a head-to-head comparison between plasma pTau181 and tau PET in a cohort of participants with subjective cognitive decline (SCD) and MCI or dementia due to AD (MCI/AD) against several clinically relevant measures. We examined their accuracies in discriminating cognitive stage (MCI/AD vs. SCD) and preclinical Aβ status (SCD Aβ-positive vs. SCD Aβ-negative), their associations with cross-sectional and longitudinal cognition, their longitudinal changes over time, and longitudinal tau biomarker relationships with longitudinal cognition.

## MATERIALS AND METHODS

### Participants

This study included all individuals from the Amsterdam Dementia Cohort and SCIENCe project with a clinical diagnosis of SCD (*n* = 50), MCI due to AD (*n* = 10), or probable AD dementia (*n* = 50) who underwent ^18^F-flortaucipir (tau) PET and had a plasma sample biobanked within 12 mo from tau PET (median, 5.0 mo; interquartile range, 4.4 mo) (20–23). The supplemental materials available at http://jnm.snmjournals.org provide details (20–24). SCD participants underwent ^18^F-florbetapir (Aβ) PET for visual assessment of Aβ status for research purposes (25). All MCI or AD dementia participants were biomarker-defined as Aβ-positive by means of abnormal cerebrospinal fluid Aβ1-42 biomarkers (according to routine thresholds (24)) or a positive Aβ PET visual read. MCI and AD dementia participants were grouped into a single MCI/AD group. The study protocol was approved by the institutional review board of the Amsterdam UMC. All participants provided written informed consent.

### Blood Sampling and Analyses

Ethylenediaminetetraacetic acid plasma samples were collected through venipuncture. A subset (*n* = 40) had 2.2 ± 0.5 y of follow-up samples available.

Samples were measured using the Simoa pTau181 V2 Advantage kit (Quanterix) on the Simoa HDx analyzer (Quanterix) (26). Samples were measured in duplicate, with an average intra-assay coefficient of variation of 6.1% ± 4.6%. One SCD participant was a clear outlier longitudinally and therefore excluded from longitudinal analyses (Supplemental Fig. 1).

### Tau PET Acquisition and Analyses

Participants underwent dual-time-point dynamic ^18^F-flortaucipir PET scans of at least 100-min duration (27*,*^28^). A subset (*n* = 40, the same subset as that with longitudinal plasma) had 2.1 ± 0.1 y of follow-up tau PET available.

We extracted nondisplaceable binding potential (BP_ND_) from 3 subject-space regions of interest (ROIs) selected a priori and corresponding to postmortem staging of neurofibrillary tangle pathology (29), in line with previous work (30*,*^31^). These ROIs included the entorhinal cortex (Braak I); a temporal composite region (Braak III and IV); and a widespread neocortical region (Braak V and VI). Details are described in the supplemental methods (27–29*,*^32^–^36^).

### Neuropsychological Assessment

Participants underwent a standardized neuropsychological assessment as part of diagnostic screening, and the assessment was repeated annually (20*,*^25^). We used neuropsychological test data from both before and after tau PET and blood collection to accurately estimate slopes in cognitive functioning. The result was longitudinal cognitive data covering 3.2 ± 2.7 y (total of 405 visits; range, 1–13; median, 3; 96 participants ≥ 2) (supplemental methods (25*,*^37^)). We a priori selected cognitive tests shown to be sensitive in capturing cognitive decline in early and late stages of AD (38): the Dutch version of the Rey Auditory Verbal Learning Test (RAVLT) delayed recall (episodic memory); the Category Fluency test (CFT) animals (semantic memory); and the Trail-Making Test B (TMT-B) (executive functioning). The Mini-Mental State Examination (MMSE) was used as a measure of global cognition.

### Statistical Analyses

We used R, version 4.0.3, for statistical analyses. A *P* value of less than 0.05 was considered significant.

Demographic characteristics were compared using *t* tests, χ^2^ tests, and Mann–Whitney *U* tests. Associations of tau markers with age, sex, and apolipoprotein E (*APOE*) ɛ4 status were examined using Pearson correlations or *t* tests. Associations between tau markers were examined using linear regressions adjusted for age, sex, and time between PET and blood collection. We examined between-group differences in tau markers using age- and sex-adjusted analysis of covariances. We performed receiver-operating-characteristic analyses to compare tau marker accuracies in discriminating cognitive stage (SCD vs. MCI/AD) and preclinical Aβ status (SCD Aβ-negative vs. SCD Aβ-positive). Differences between areas under the curve (AUCs) were tested using DeLong tests.

Next, we investigated associations of tau markers with cognitive decline using age-, sex-, and education-adjusted linear mixed models (LMMs) with subject-specific intercepts. For all LMMs, a random slope was added when it improved model fit by comparing the Akaike information criterion using χ^2^ statistics. Tau marker (tau PET or plasma pTau181), time (tau PET or blood collection as T = 0), and an interaction term of tau marker × time were entered as fixed variables and neuropsychological test performance as a dependent variable. For all LMMs, we used separate models per tau marker and per cognitive test. Furthermore, tau markers and cognitive scores were scaled within each LMM to compare effect sizes. The fixed effect of tau marker was interpreted as the cross-sectional association, and the fixed effect of tau marker × time was interpreted as the longitudinal association. *P* values were corrected for multiple testing by applying the 10% false-discovery rate (FDR).

Lastly, in the subset with longitudinal tau markers, we investigated changes in tau markers over time using age- and sex-adjusted LMMs. Time, diagnosis (SCD or MCI/AD), and an interaction term of diagnosis × time were entered as fixed variables and tau marker as a dependent variable. We additionally explored associations of tau marker annual changes with cognitive decline, for which tau marker annual changes were calculated as [(follow-up − baseline)/time between measurements in years]. Age-, sex-, and education-adjusted LMMs were performed with tau marker annual change, time (baseline tau PET or blood collection as T = 0), and an interaction term of tau marker annual change × time as a fixed variable and neuropsychologic test performance as a dependent variable. The fixed effect of tau marker annual change × time was interpreted as the association between tau marker annual change and longitudinal cognition.

## RESULTS

### Participants

Table 1 shows the participant characteristics. Mean age was 65.4 ± 7.4 y, and 48.2% of participants were female. By study design, all MCI/AD participants were Aβ-positive. Of the 50 SCD participants, 19 (38.0%) were Aβ-positive. There were no group differences in age, sex, or education, but there were more *APOE* ɛ4 carriers in MCI/AD than in SCD (*P* < 0.01). Supplemental Table 1 shows the characteristics of the longitudinal subset. Plasma pTau181 did not correlate with age or sex in either SCD or MCI/AD. In SCD, but not MCI/AD, *APOE* ɛ4 carriers showed higher plasma pTau181 than noncarriers (*P* = 0.03). Tau PET BP_ND_ in the temporal ROI positively correlated with age in SCD (*r* = 0.29, *P* = 0.04), whereas in MCI/AD, tau PET BP_ND_ in all ROIs negatively correlated with age (−0.37 > *r* > −0.62; all ROIs, *P* < 0.01). In MCI/AD, but not SCD, female participants showed higher BP_ND_ than male participants (all ROIs *P* < 0.01). In both SCD and MCI/AD, *APOE* ɛ4 carriers showed higher entorhinal tau PET BP_ND_ than noncarriers (*P* = 0.01 and *P* = 0.03, respectively) but not in other ROIs (Supplemental Table 2; Supplemental Fig. 2).

**TABLE 1.** Demographics

		Stratified by diagnosis	
Demographic	Total sample	SCD	MCI/AD
Participants (*n*)	110 (100%)	50 (45.5%)	60 (55.5%)
Age (y)	65.4 ± 7.4	65.6 ± 7.6	65.3 ± 7.3
Sex, female (*n*)	53 (48.2%)	25 (50.0%)	28 (46.7%)
Median education	6 (range, 2–7)	6 (range, 2–7)	6 (range, 3–7)
*APOE* ɛ4 carrier (*n*)	61 (56.5%)	18 (37.5%)	43 (71.7%)*
Aβ-positive status (*n*)	79 (71.8%)	19 (38.0%)	60 (100%)*
Cognition			
MMSE	25.7 ± 4.2	28.8 ± 1.4	23.0 ± 4.0*
RAVLT delayed recall	5.7 ± 4.4	9.3 ± 3.3	2.8 ± 2.8*
CFT animals	19.9 ± 7.3	24.8 ± 5.7	15.6 ± 5.7*
Trail-making test B	122.5 ± 83.7	78.0 ± 35.8	174.3 ± 93.4*
Plasma pTau181 (pg/mL)	2.53 ± 1.14	2.08 ± 1.17	2.91 ± 0.98*
^18^F-flortaucipir PET BP_ND_			
Entorhinal	0.16 ± 0.26	−0.03 ± 0.17	0.32 ± 0.21*
Temporal	0.32 ± 0.31	0.10 ± 0.15	0.50 ± 0.29*
Neocortical	0.22 ± 0.29	0.05 ± 0.07	0.36 ± 0.33*

* Different from SCD at *P* < 0.01.

Data are mean ± SD unless specified otherwise. Education reflects Dutch Verhage scale. APOE E4 was missing in 2 SCD patients, CFT animals was missing in 3 MCI/AD patients, and Trail-making test B was missing in 17 MCI/AD patients.

### Association Between Plasma pTau181 and Tau PET

**FIGURE 1.**
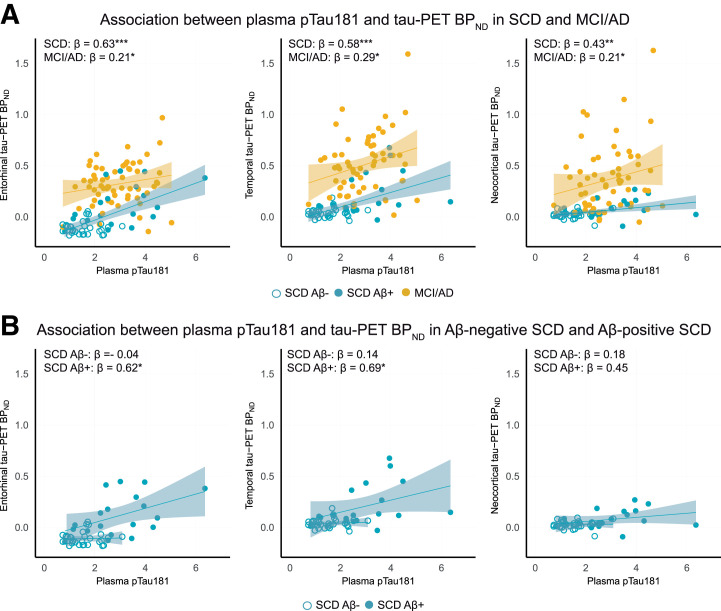
Associations between plasma pTau181 and tau PET in SCD and MCI/AD (A) and SCD Aβ-negative and SCD Aβ-positive (B) participants. *P < 0.5. **P < 0.01. ***P < 0.001.

Across all participants, plasma pTau181 was associated with tau PET in each ROI (range of β, 0.37–0.53; all *P* < 0.01) (Supplemental Tables 3 and 4). Within SCD and MCI/AD separately, plasma pTau181 was associated moderately with tau PET in SCD (range of β, 0.43–0.63; all *P* < 0.01) and associated weakly to moderately with tau PET in MCI/AD (range of β, 0.21–0.29; all *P* < 0.05) (Fig. 1A). Further stratifying SCD participants for Aβ positivity revealed significant positive associations between plasma pTau181 and tau PET in SCD Aβ-positive participants but not in SCD Aβ-negative participants (Fig. 1B).

### Comparing Plasma pTau181 and Tau PET for Predicting Cognitive Stage and Preclinical Aβ status

**FIGURE 2.**
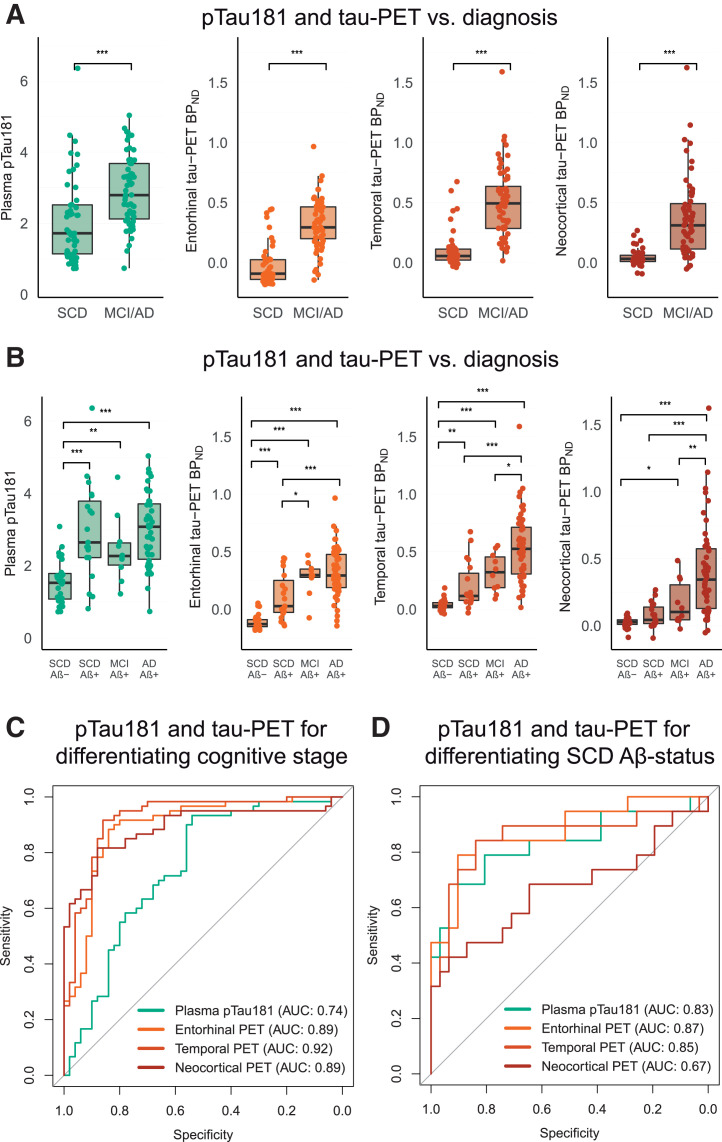
(A and C) Plasma pTau181 and tau PET BP_ND_ stratified for SCD and MCI/AD (A) and for SCD Aβ-negative and SCD Aβ-positive MCI due to AD and AD dementia (C). (B and D) AUCs for discriminating SCD from MCI/AD (B) and SCD Aβ-negative from SCD Aβ-positive (D) participants. *P < 0.5. **P < 0.01. ***P < 0.001.

Both plasma pTau181 and tau PET BP_ND_ were higher in MCI/AD than in SCD (all *P* < 0.001), although plasma pTau181 showed considerable between-group overlap (Fig. 2A). The AUC for distinguishing MCI/AD from SCD for plasma pTau181 (AUC, 0.74 [95% CI, 0.65–0.84]) was significantly lower than that for tau PET BP_ND_ in entorhinal (0.89 [95% CI, 0.83–0.96], DeLong *P* < 0.001), temporal (0.92 [95% CI, 0.87–0.98], *P* < 0.001), and neocortical (0.89 [95% CI, 0.83–0.95], *P* = 0.005) ROIs (Fig. 2C).

When the cohort was stratified into SCD Aβ-negative, SCD Aβ-positive, MCI, and AD dementia groups, plasma pTau181 was higher in each Aβ-positive group (SCD Aβ-positive, MCI, and AD dementia) than in Aβ-negative SCD (Fig. 2B; Supplemental Table 5). No differences were observed between Aβ-positive groups. In contrast, tau PET showed more stepwise increases across groups (Fig. 2B; Supplemental Table 5).

Finally, to distinguish preclinical Aβ status (SCD Aβ-positive vs. SCD Aβ-negative), plasma pTau181 showed an AUC of 0.83 (95% CI, 0.70–0.96). Comparable AUCs were observed for tau PET BP_ND_ in entorhinal (0.87 [95% CI, 0.77–0.98], *P* = 0.54), temporal (0.85 [95% CI, 0.73–0.98], *P* = 0.80), and neocortical (0.67 [95% CI, 0.50–0.84], *P* = 0.09) regions (Fig. 2D).

### Comparing Plasma pTau181 and Tau PET for Predicting Cognitive Decline

Next, we investigated associations with cross-sectional and longitudinal cognition. We report associations for plasma pTau181 and temporal tau PET in SCD and MCI/AD that survived FDR correction. Supplemental Table 6 reports all estimates and uncorrected *P* values.

**FIGURE 3.**
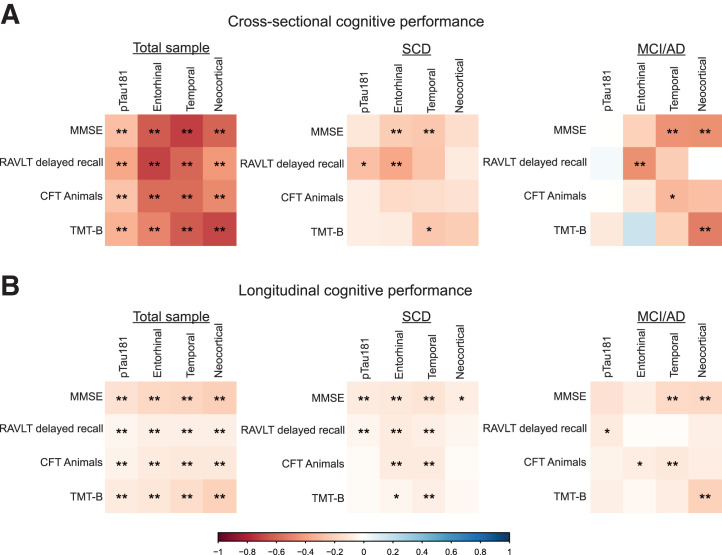
Heat plots reflecting standardized β-estimates (color scale) and significance levels from LMMs between plasma pTau181 or tau PET (predictor) and cross-sectional (A) and longitudinal (B) cognitive performance (outcome variables) (age-, sex-, and education-adjusted). *Uncorrected *P* < 0.05. **FDR *P* < 0.05. TMT-B = trail-making test B.

In SCD, plasma pTau181 was not associated with cross-sectional performance on any of the included neuropsychological tests (FDR *P* > 0.05). Longitudinally, higher plasma pTau181 was associated with a steeper rate of decline on the MMSE (β = −0.05, FDR *P* < 0.01) and RAVLT delayed recall (β = −0.08, FDR *P* = 0.04). In SCD, temporal tau PET BP_ND_ was associated with worse cross-sectional performance on the MMSE (β = −0.24, FDR *P* = 0.04). In addition, temporal tau PET BP_ND_ was associated with a steeper rate of decline on all neuropsychologic tests (MMSE: β = −0.12, FDR *P* < 0.01; RAVLT delayed recall: β = −0.07, FDR *P* = 0.01; CFT animals: β = −0.08, FDR *P* < 0.01; trail-making test B: β = −0.07, FDR *P* < 0.01) (Fig. 3).

In MCI/AD, plasma pTau181 was not associated with cross-sectional or longitudinal performance on any of the included neuropsychological tests (FDR *P* > 0.05). In contrast, in MCI/AD, temporal tau PET BP_ND_ was associated with worse cross-sectional performance on the MMSE (β = −0.45, FDR *P* < 0.01) and with a steeper rate of decline on the MMSE (β = −0.17, FDR *P* < 0.01) and on the CFT animals test (β = −0.10, FDR *P* = 0.04) (Fig. 3).

### Comparing Longitudinal Changes in Plasma pTau181 and Tau PET

**FIGURE 4.**
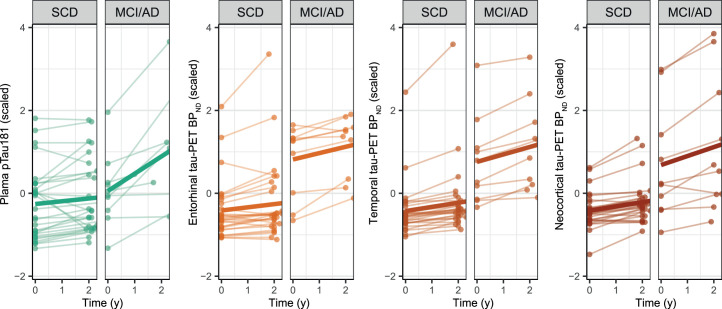
Spaghetti plots of scaled longitudinal plasma pTau181 and tau PET in SCD and MCI/AD.

Finally, in the subset with repeated tau biomarker assessments, an interaction effect of diagnosis × time was observed for plasma pTau181 (β = 0.35, *P* < 0.001), meaning plasma pTau181 levels increased more steeply in MCI/AD than in SCD (Fig. 4). For tau PET, we also observed significant interaction effects of diagnosis × time, with steeper increases in BP_ND_ in MCI/AD than in SCD in temporal (β = 0.08, *P* = 0.049) and neocortical (β = 0.12, *P* < 0.02), but not entorhinal (β = 0.08, *P* = 0.14), regions (Fig. 4). Supplemental Table 7 reports longitudinal changes in tau markers in SCD and MCI/AD separately.

Annual change in plasma pTau181 was not associated with longitudinal cognition (all *P* > 0.05). In contrast, annual change in tau PET BP_ND_ in all ROIs was associated with decline on the RAVLT delayed recall (all *P* < 0.05) (Supplemental Fig. 3). Furthermore, annual change in temporal and neocortical BP_ND_ was associated with decline on the CFT animals, and neocortical BP_ND_ additionally was associated with decline on the MMSE (Supplemental Table 8 shows estimates and *P* values).

## DISCUSSION

In this study, we performed a head-to-head comparison between plasma pTau181 and tau PET in predicting cognitive stage, preclinical Aβ status, and cross-sectional and longitudinal cognitive functioning. Both plasma pTau181 and tau PET discriminated with high accuracy between SCD Aβ-negative and SCD Aβ-positive individuals, but tau PET outperformed plasma pTau181 in discriminating cognitive stage (MCI/AD vs. SCD). Moreover, compared with plasma pTau181, tau PET showed stronger associations with cognitive decline and was associated with a wider variety of cognitive tests. Both plasma pTau181 and tau PET showed steeper increases over time in MCI/AD than in SCD, but only annual changes in tau PET were associated with longitudinal decline. Our results provide support for both plasma pTau181 and tau PET as biomarkers for identifying Aβ pathology but indicate that tau PET has better performance for disease staging and clinical progression.

For distinguishing between preclinical Aβ-positive and Aβ-negative individuals, plasma pTau181 and tau PET (especially in entorhinal and temporal regions) showed high accuracy and performed equally well (AUCs of 0.83–0.87). This finding highlights the close relationship of both plasma pTau181 and ^18^F-flortaucipir PET with the presence of Aβ pathology and underscores the ability of these markers to predict Aβ status even at a very early stage, in line with previous studies (7*,*^8^*,*^14^*,*^19^). Combined with the practical advantages of plasma biomarkers, our results support the potential of plasma pTau181 for implementation in the clinic as a first step in the diagnostic work-up of AD or as a clinical trial screening or prescreening tool, before cerebrospinal fluid or PET measurements.

For distinguishing between cognitively impaired and unimpaired individuals, tau PET significantly outperformed plasma pTau181 (AUCs of 0.89–0.92 for tau PET vs. 0.74 for pTau181). A stronger role for tau PET than for plasma pTau181 in disease staging was further strengthened by the comparison of tau marker values between SCD Aβ-negative, SCD Aβ-positive, MCI, and AD dementia, which showed stepwise increases in tau PET binding across the groups, whereas no differences in plasma pTau181 were observed among the Aβ-positive groups of different cognitive stages. In addition, tau PET associations with cross-sectional and longitudinal cognitive functioning were stronger and involved a wider variety of cognitive tests than was observed for plasma pTau181. The observed differences between the tau markers for predicting cognition might be related to biologic differences. Whereas fluid tau markers reflect increased phosphorylation and release of soluble tau (39), tau PET tracers bind to insoluble tau aggregates. Strong associations between tau tracer binding, disease stage, and cognitive decline have also been observed in previous studies (17*,*^40^). Overall, our results provide stronger support for tau PET than for plasma pTau181 for tracking disease progression and for use as a potential prognostic biomarker and clinical trial outcome measure.

Our longitudinal analyses showed that both plasma pTau181 and tau PET show steeper increases over time in MCI/AD than in SCD, in line with previous studies and with similar magnitudes (41–43). However, annual increases in only tau PET, not plasma pTau181, were associated with cognitive decline. A previous study investigating plasma pTau217 did observe associations between annual plasma pTau217 changes and longitudinal cognition (42). This discrepancy could be related to a different plasma pTau isoform or assay (26), and although our longitudinal results should be interpreted with caution because of the small sample size, our finding warrants further investigation as it could have implications for clinical trial designs. Previous studies have suggested that plasma pTau217 might have slightly favorable properties compared with plasma pTau181 in terms of dynamic range (44), prediction of Aβ status (10), and differentiation between clinical AD dementia and other neurodegenerative dementias (45). However, comparable performance for pTau181 and pTau217 has also been observed, such as in differentiating AD dementia from controls (10*,*^26^). Head-to-head comparisons including different plasma pTau isoforms are needed to define the complementarity of these markers.

This study had some limitations. Our cohort consisted of a highly selected sample with a relatively high percentage of Aβ-positive SCD cases. Head-to-head comparisons between plasma pTau181 and tau PET in unselected cohorts, more diverse populations, and non-AD dementias would be important. Furthermore, we had a relatively small sample size in longitudinal analyses. In addition, we used plasma pTau181 and ^18^F-flortaucipir PET, but studies have shown that other plasma pTau isoforms and second-generation PET tracers may be more sensitive for earlier disease stages (26*,*^29^*,*^45^). Finally, a recent study showed that health conditions such as chronic kidney disease, hypertension, stroke, and myocardial infarction are associated with plasma pTau181 (46). Future studies with larger sample sizes are needed to further investigate this possibility.

## CONCLUSION

Plasma pTau181 and tau PET performed equally well in identifying Aβ pathology, but tau PET better monitored disease stage and clinical progression.

## DISCLOSURE

Alzheimer Center Amsterdam is supported by Stichting Alzheimer Nederland and Stichting VUmc fonds. This study was made possible by ZonMW Memorabel, Dioraphte, Avid Radiopharmaceuticals, and Janssen Pharmaceuticals. Albert Windhorst is editor-in-chief of *Nuclear Medicine & Biology*. Philip Scheltens receives consultancy fees (paid to the university) from AC Immune, Alzheon, Brainstorm Cell, ImmunoBrain Checkpoint, Novartis, and Novo Nordisk; is a principal investigator (within university affiliation) of studies with AC Immune, FUJI-film/Toyama, IONIS, UCB, and Vivoryon; and is an employee of Life Sciences Partners Amsterdam. Wiesje van der Flier receives grant support from ZonMW, NWO, EU-FP7, EU-JPND, Alzheimer Nederland, Hersenstichting CardioVascular Onderzoek Nederland, Health∼Holland, Topsector Life Sciences & Health, Stichting Dioraphte, Gieskes-Strijbis Fonds, Stichting Equilibrio, Pasman Stichting, Stichting Alzheimer & Neuropsychiatrie Foundation, Philips, Biogen MA Inc., Novartis-NL, Life-MI, AVID, Roche BV, Fujifilm, and Combinostics; holds the Pasman chair; is a recipient of ABOARD (a public–private partnership receiving funding from ZonMW [grant 73305095007] and Health∼Holland, Topsector Life Sciences & Health [PPP-allowance LSHM20106]); performs contract research for Biogen MA Inc. and Boehringer Ingelheim; is an invited speaker for Boehringer Ingelheim, Biogen MA Inc., Danone, Eisai, WebMD Neurology (Medscape), and Springer Healthcare; is a consultant to Oxford Health Policy Forum CIC, Roche, and Biogen MA Inc.; is on the advisory boards of Biogen MA Inc. and Roche, with all funding paid to her institution; was associate editor of *Alzheimer’s Research & Therapy*; and is associate editor at *Brain*. Bart van Berckel receives research support from EU-FP7, CTMM, ZonMw, NOW, and Alzheimer Nederland; performed contract research for Rodin, IONIS, AVID, Eli Lilly, UCB, DIAN-TU, and Janssen; was a speaker at a symposium organized by Springer Healthcare; has a consultancy agreement with IXICO for PET visual readings; is a trainer for GE; and receives financial compensation only from Amsterdam UMC. Charlotte Teunissen is on the European Commission (Marie Curie International Training Network, grant 860197 [MIRIADE], and JPND), Health Holland, the Dutch Research Council (ZonMW), the Alzheimer Drug Discovery Foundation, the Selfridges Group Foundation, Alzheimer Netherlands, and the Alzheimer Association; is a recipient of ABOARD (a public–private partnership receiving funding from ZonMW [grant 73305095007] and Health∼Holland, Topsector Life Sciences & Health [PPP-allowance LSHM20106]); has collaboration contracts with ADx Neurosciences, Quanterix, and Eli Lilly; performs contract research for AC-Immune, Axon Neurosciences, Biogen, Brainstorm Therapeutics, Celgene, EIP Pharma, Eisai, PeopleBio, Roche, Toyama, and Vivoryon; and is on editorial boards for Medidact Neurologie/Springer, *Alzheimer’s Research & Therapy,* and *Neurology: Neuroimmunology & Neuroinflammation*. No other potential conflict of interest relevant to this article was reported.

## Notes

KEY POINTS **QUESTION:** How do cross-sectional and longitudinal plasma pTau181 and tau PET perform in predicting cognitive stage, preclinical Aβ status, and longitudinal cognition? **PERTINENT FINDINGS:** Both plasma pTau181 and tau PET discriminated Aβ-negative from Aβ-positive cognitively unimpaired individuals with high accuracy. Tau PET outperformed plasma pTau181 in discriminating cognitively impaired from unimpaired individuals and in predicting cognitive decline. Both plasma pTau181 and tau PET showed steeper longitudinal increases in cognitively impaired than unimpaired individuals, but only annual changes in tau PET were associated with longitudinal cognitive decline. **IMPLICATIONS FOR PATIENT CARE:** Both plasma pTau181 and tau PET can be used for predicting Aβ status, but tau PET better monitors disease stage and predicts cognitive decline.
